# Supplementary material for: Identification of photoperiod-induced specific miRNAs in the adrenal glands of Sunite sheep (Ovis aries)
Source: Front Vet Sci. 2022 Jul 22;9:888207. doi: 10.3389/fvets.2022.888207 (PMC9354845; doi:10.3389/fvets.2022.888207)
Supplement: Supplementary file 1 [file Data_Sheet_1.ZIP › Supplementary file/miRDeep2 file.zip!/miRDeep2 file/Novel_Structure/SP42A3/Novel_641.pdf]

Provisional ID	:	NC_019470.2_20077
Score total	:	13
Score for star read(s)	:	3.9
Score for read counts	:	5.7
Score for mfe	:	1.7
Score for randfold	:	1.6
Score for cons. seed	:	
Total read count	:	23
Mature read count	:	22
Loop read count	:	0
Star read count	:	1

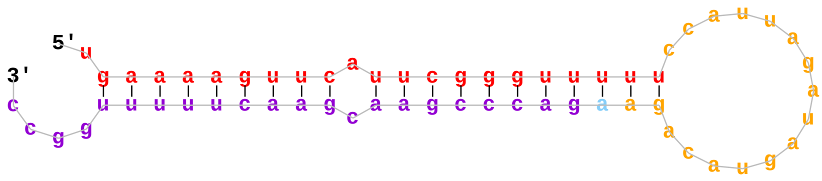

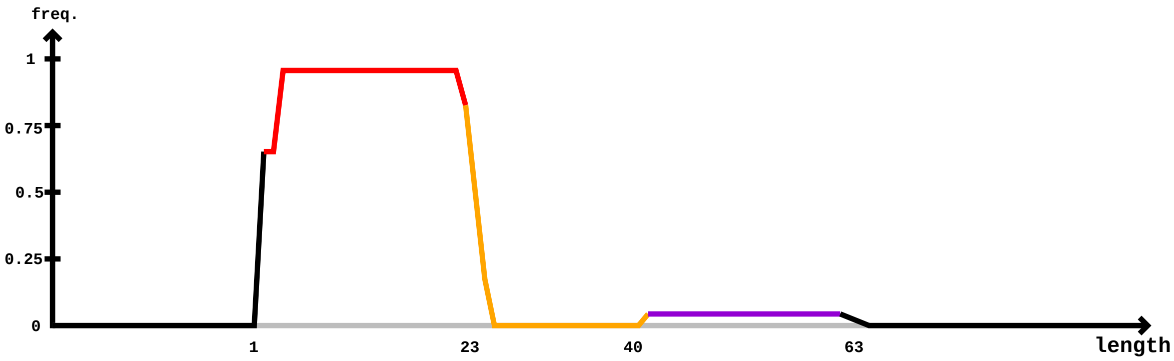

Mature Star

```
5'- cugccuucuauugggcuggcugaaaaguucauucggguuuuuccauuagauaguacagaagacccgaacgaacuuuuuggccaaccaauacauuuuccaucucuguucuucc   -3'    obs
    cugccuucuauugggcuggcugaaaaguucauucggguuuuuccauuagauaguacagaagacccgaacgaacuuuuuggccaaccaauacauuuuccaucucuguucuucc          exp
    ........((((((..((((((((((((((.(((((((((((...............))))))))))).)))))))))))))).)))))).......................   reads   mm   sample
    ....................ugaaaaguucauucggguuuu.......................................................................   3       0    seq
    ....................ugaaaaguucauucggguuuuu......................................................................   12      0    seq
    ......................aaaaguucauucggguuuuu......................................................................   1       0    seq
    ......................aaaaguucauuAggguuuuuc.....................................................................   2       1    seq
    ......................aaaaguucauuAggguuuuucc....................................................................   4       1    seq
    ............................................................gacccgaacgaacuuuuuggcc..............................   1       0    seq
```
